# Moving toward elucidating alternative motor pathway structures post-stroke: the value of spinal cord neuroimaging

**DOI:** 10.3389/fneur.2024.1282685

**Published:** 2024-02-14

**Authors:** Ramiro Oquita, Victoria Cuello, Sarvani Uppati, Sravani Mannuru, Daniel Salinas, Michael Dobbs, Kelsey A. Potter-Baker

**Affiliations:** ^1^School of Medicine, University of Texas Rio Grande Valley, Edinburg, TX, United States; ^2^Department of Neuroscience, School of Medicine, University of Texas Rio Grande Valley, Edinburg, TX, United States; ^3^Department of Clinical Neurosciences, College of Medicine, Florida Atlantic University, Boca Raton, FL, United States

**Keywords:** stroke, alternate motor pathways, reticulospinal, rubrospinal, imaging

## Abstract

Stroke results in varying levels of motor and sensory disability that have been linked to the neurodegeneration and neuroinflammation that occur in the infarct and peri-infarct regions within the brain. Specifically, previous research has identified a key role of the corticospinal tract in motor dysfunction and motor recovery post-stroke. Of note, neuroimaging studies have utilized magnetic resonance imaging (MRI) of the brain to describe the timeline of neurodegeneration of the corticospinal tract in tandem with motor function following a stroke. However, research has suggested that alternate motor pathways may also underlie disease progression and the degree of functional recovery post-stroke. Here, we assert that expanding neuroimaging techniques beyond the brain could expand our knowledge of alternate motor pathway structure post-stroke. In the present work, we will highlight findings that suggest that alternate motor pathways contribute to post-stroke motor dysfunction and recovery, such as the reticulospinal and rubrospinal tract. Then we review imaging and electrophysiological techniques that evaluate alternate motor pathways in populations of stroke and other neurodegenerative disorders. We will then outline and describe spinal cord neuroimaging techniques being used in other neurodegenerative disorders that may provide insight into alternate motor pathways post-stroke.

## 1 Introduction

Stroke is a leading cause of serious, long-term disability in the world (1). Common consequences of stroke include paralysis, gait dysfunction, sensory deficits, speech impairments, cognitive impairments, pain, and depression, with ~90% of patients experiencing persistent neurological motor deficits that lead to disability and handicaps (2). Damage to and subsequent degeneration of motor pathways contribute significantly to motor impairment. Indeed, evidence has been established dating back to 1901 when Barnes (3) observed degeneration in the spinal cord in postmortem analyses of hemiplegia. In fact, early analyses of tracts in the 20th century were predominantly based on postmortem examinations of degeneration resulting from clinical cases or preclinical studies in animals (4–6). Today, modern neuroimaging techniques, such as diffusion tensor imaging, allow for non-invasive investigation of the integrity of motor pathways in living stroke patients. As a result, numerous research groups have established degeneration in the corticospinal tract (CST) as a significant contributor to motor impairment and recovery prognosis (7–11). For example, through the use of diffusion tensor imaging, Maraka et al. (9) found a significant correlation between the degree of damage to the corticospinal tract and the severity of motor impairment in patients in each phase of ischemic stroke. In addition, Byblow et al. (12) have further suggested that acute stroke patients show ~70% resolution of motor impairment if corticospinal tracts are functional. Overall, neuroimaging studies have confirmed that amount, connectivity, and neurophysiologic function of CST is related to impairment and functional recovery post-stroke (13–16).

Work has also suggested that alternate motor pathways such as the lateral and medial reticulospinal tracts, descending medial longitudinal fasciculus, and rubrospinal tract may also impact baseline motor function post-stroke (17, 18) and motor recovery (19–21). However, the role and function of alternate motor pathways have not been thoroughly evaluated in the context of post-stroke recovery, particularly in human subjects. This is largely since most work to date has evaluated the brain, which has a unique anatomical organization. Specifically, the descending and ascending alternate motor pathways can be difficult to distinguish with brain imaging techniques because these pathways overlap in the brain or originate caudal to the pons. For example, several groups have been able to utilize advanced neuroimaging techniques to delineate some alternate motor pathways, such as the corticoreticular pathway (CRP), in the brain in healthy individuals and with various neuropathologies (22–25), but brain stem or pathways in the spinal cord implicated in stroke recovery are less understood.

Thus, we assert that since several alternate motor pathways begin to delineate as the tracts progress into the spinal cord or originate subcortically, that future efforts should consider the spinal cord as a future focus area. Specifically, several studies have suggested a role of alternate motor pathways post-stroke using *in vivo* studies, electrophysiological methods or magnetic resonance imaging targeting the brain (17, 19, 26–28). Here we suggest that by using existing or creating new techniques, such as high-resolution magnetic resonance imaging (MRI) or spinal cord analyses that focus on areas caudal to the brain, such as the brainstem or spinal cord, the structures of alternate motor pathways can be visualized and knowledge gained may enhance treatment modalities post-stroke (17). Thus, in the present article, we will first briefly review studies that suggest the role of alternate motor pathways in stroke impairment and recovery and the electrophysiological techniques that have been used to evaluate alternate motor pathways. Then as a primary focus, we will then provide examples of visualization and quantification methods that may be used to expand our current knowledge of alternate motor pathways in future clinical studies in populations with stroke.

## 2 Evaluation of alternate motor pathway role after stroke: *in vivo* and clinical studies

**Figure 1:**
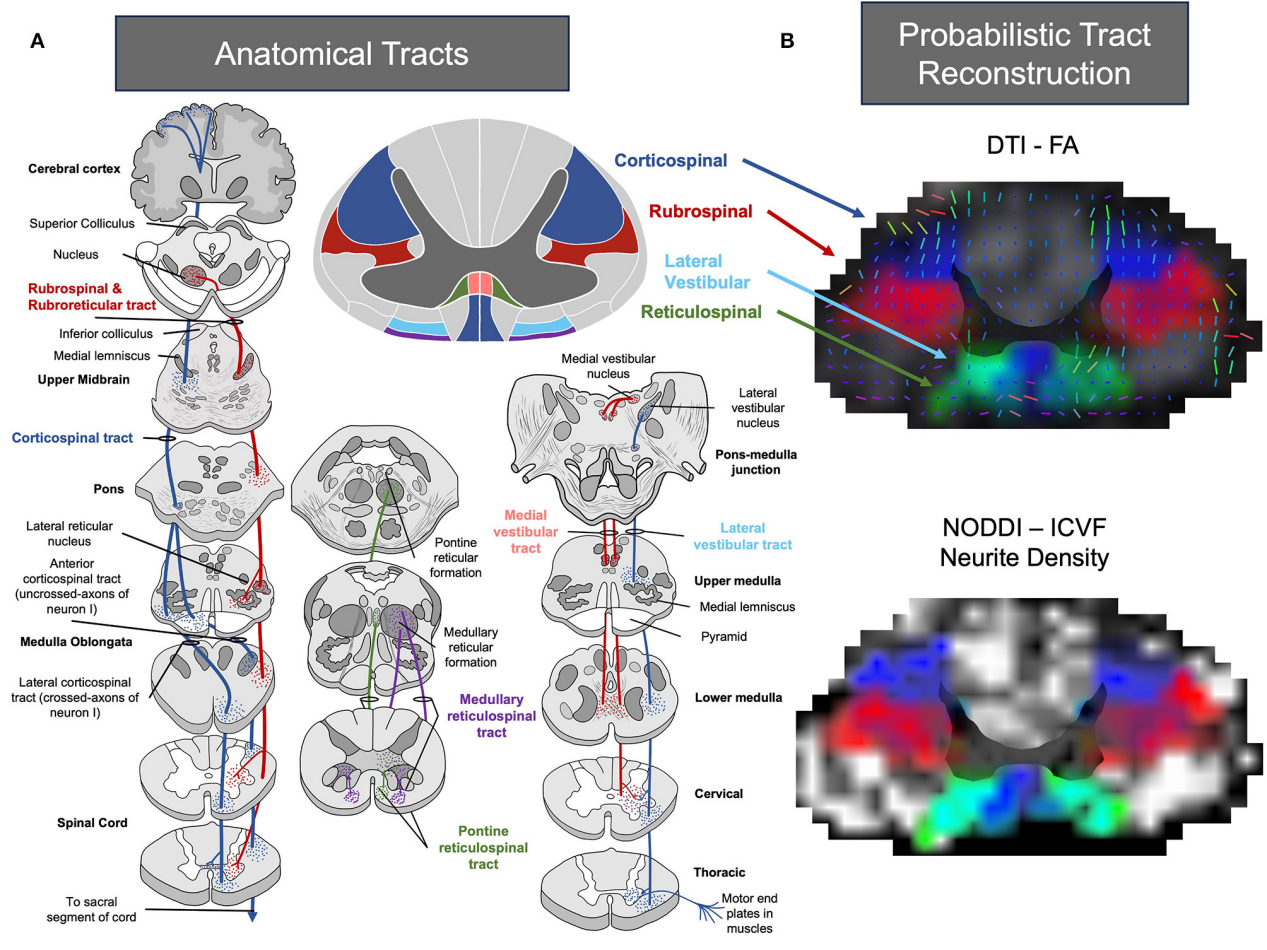
**(A)** Pictural representation of common tracts associated with recovery following stroke. Image representations were adapted from (35–38). **(B)** Atlas-based probabilistic tract reconstruction utilizing FA and Intra-Cellular Volume Fraction (ICVF) with the Spinal Cord Toolbox for visualizing alternate motor pathways throughout the spinal cord (35, 36, 39). Images of the cervical region were processed using linear interpolation, and the alpha parameter was modified based on tract intensity.

Several *in vivo*, clinical and post-mortem studies have sought to determine the role of alternate motor pathways after stroke (29–34). The pathways that have received the most attention have included the reticulospinal, rubrospinal, and vestibulospinal. Depictions of the most evaluated alternate motor pathways after stroke are shown in Figure 1A. In general, literature supports the idea that while alternate motor pathways have a compensatory role in post-stroke recovery, the ipsilesional corticospinal tract remains critical for fine motor control (30). In contrast, alternate motor pathways have been shown to have more of a compensatory role (20), often involved in synergistic movements (40), and are mostly implicated in gross movement or proximal muscle function. Here, we will first briefly review the identified roles of these pathways post-stroke, as a detailed review of each pathway post-stroke is outside the scope of the present work. For a detailed scope of reticulospinal pathways (41–43), please refer to the following reviews and studies (44–46). For a detailed scope of rubrospinal pathways, please refer to the following reviews and studies (47–52). For a detailed scope of vestibulospinal pathways, please refer to the following reviews and studies (43, 53–57).

### 2.1 Reticulospinal pathways

The reticulospinal pathways emerge from the pontomedullary reticular formation, located in the midbrain, and project bilaterally in the spinal cord (58) (Figure 1A). The reticulospinal pathways have been implicated in posture (59), upper limb flexor movement, and locomotion (58, 60–64). In addition, studies have suggested that the reticulospinal pathways work in tandem with the corticospinal pathways to facilitate control of skilled reaching (65–69). Indeed, hallmark studies by Drew (65–69) in intact cats have shown a direct role of reticulospinal pathways in voluntary gait, postural control, and bilateral reaching movements. Overall, the reticulospinal tract has been indicated to facilitate motor function mainly in proximal muscles (up to 40% control). Although, the projections of the reticulospinal tract may also indicate a role in recovery of the lower limb in stroke (70).

Given this role, it is not surprising that studies have begun to evaluate the possible influence of reticulospinal pathways in stroke recovery. In general, the majority of work to date has focused on the role of the reticulospinal pathway in the upper limb. For example, in a macaque model, lesions to the corticospinal tract were found to result in an increase in activity in intact ipsilateral reticulospinal pathways on the non-lesioned hemisphere of the brain (61). Hebert et al. (61) also observed that increases in ipsilateral reticulospinal pathway activity correlated with reaching function. Zaaimi et al. (29) built on this work by evaluating extracellular reticular spikes in macaques that were healthy or 1 year post pyramidal tract lesion. The group observed that after a pyramidal tract lesion, membrane properties in the reticular nuclei changed and resulted in increased reticulospinal output. It was suggested that such change may help compensate for the loss of drive from the corticospinal pathways (29). Similar observations were noted by Darling et al. (19). Specifically, their group observed that recovery after motor cortex injury was correlated with projections to the reticular nuclei from the non-lesioned hemisphere.

Additional studies have confirmed the role of the reticulospinal pathway in clinical populations, although in general, data is limited (17, 63, 64, 71–74). For example, similar to Hebert et al. (61), Karbasforoushan et al. (17) observed that individuals with a chronic unilateral internal capsule stroke, who had significant damage to the corticospinal pathway, demonstrated higher white matter integrity in ipsilateral projections to the paretic limb in the medial reticulospinal tract compared to healthy controls. It has also been noted that patients with a more intact medial reticulospinal tract show improved motor recovery post-stroke. Work by Karbasforoushan et al. (17) was expanded upon by a recent study evaluating patients in several phases of ischemic stroke recovery. Specifically, Choudhury et al. (75) evaluated patients presenting 6 months to 12 years post-stroke and identified that reticulospinal tract pathways were critical to regain arm and hand function in patients with severe damage to the corticospinal tract. Further, recent reports have even suggested that intervention approaches targeting the reticulospinal tract post-stroke can result in significant improvements in upper-limb function (76). Taken collectively, it has been suggested that changes in the ipsilateral reticulospinal pathways after stroke impact recruitment of the non-lesioned hemisphere of the brain and corticoreticular pathway (44) and relate to baseline impairment (17, 63, 64, 71–74) and spasticity (77–79). Although limitations should be considered, including how animal models of stroke may differ from clinical presentations, especially with respect to overall anatomical differences, chronicity (70) and methodology of the stroke lesion. In addition, recent evidence has suggested that the reticulospinal tract may have inputs to the primary and supplementary motor cortex, so it remains unclear if the role of reticulospinal tracts is of cortical or subcortical origin (58). Further, despite the influence of reticulospinal tracts in posture and locomotion in animal models, very little work has been done to evaluate the influence of these pathways in the lower limb. In addition, it remains unclear when reticulospinal pathways begin to be recruited or modified after the initial stroke event. Although some recent work has suggested that reticulospinal tract connectivity may not influence skill-acquisition gains in the sub-acute period (80).

### 2.2 Vestibulospinal pathways

The vestibulospinal pathway originates in the brain stem (Figure 1A) and contributes to the movement of intercostal muscles, back muscles, and limb extensors via unilateral and bilateral projections to the vestibulospinal reflexes (43, 54, 55, 81, 82). Hallmark work by Wilson, Peterson, and others (56, 83–85) in animal models have indicated that the vestibulospinal pathways have a critical role in body equilibrium, head position and clear vision with movement. In clinical work, vestibulospinal tracts have been linked to post-stroke spasticity (86), balance (87), vertigo/dizziness (88), and locomotion in stroke patients (89). However, similar to lower limb studies of the reticulospinal tract in populations with stroke, there currently is a lack of work evaluating the role of the vestibulospinal tract in populations with stroke. This is particularly surprising, given the growth of studies evaluating vestibular rehabilitation approaches in neurodegenerative populations (90, 91) and high prevalence of stroke patients with disturbances to the vestibular system (92, 93). Ultimately, additional studies are needed to understand the full extent of the effects of the vestibulospinal pathways following a stroke.

### 2.3 Rubrospinal pathways

Rubrospinal pathways are believed to originate from the red nucleus (49, 50) (Figure 1A) and contribute to reaching and grasping movements (49, 63). As shown in Figure 1A, the rubrospinal pathway neighbors the corticospinal tract in the majority of the spinal cord and both run in parallel. Work in animal and non-human primates has shown that rubrospinal pathways are important in the recovery of hand function after insult to the corticospinal pathway. For example, work by Bolzoni et al. (27) found that the effects of non-invasive brain stimulation to improve motor function were facilitated by both corticospinal and rubrospinal pathways in a cat model. An innovative study using anterograde tracing by Isa and colleagues also found that in rats following a lesion to the internal capsule, robust axonal sprouting from the forelimb area to the red nucleus could be observed. Isa confirmed that selective blockade of the rubrospinal pathways directly influenced reaching movements following the lesion (94). In addition, work by Cheney et al. (49, 51, 95) have also shown that following a unilateral lesion of the pyramidal tract, post-lesion reorganization occurs in the red nucleus that is linked to output of flexor muscles.

The impact of the rubrospinal pathway has also been studied in human patients with stroke (26, 27, 96, 97). For example, Rüber et al. (26) observed that red nuclei contained more gray and white matter in patients post-stroke, and that this finding was correlated with baseline hand and arm function. However, while a role for rubrospinal pathways has been proposed, current work to date has evaluated a sample of both acute and chronic patients (range 5–359 months post-stroke). Further, since most projections from the red nucleus have limited range in the spinal cord in humans (98), it has been suggested that overall influence of rubrospinal pathways may be limited in stroke recovery. Therefore, while rubrospinal pathways may have a role in stroke recovery, a deeper understanding of whether these pathways are modified in the acute or chronic phase is still required.

## 3 Electrophysiology methods to understand alternative motor pathways

Neurophysiologists have begun to expand our knowledge of the functional integrity of alternative motor pathways in animal and clinical populations. Of note, use of the startle reflex, transcranial magnetic stimulation, long latency responses (28) and galvanic stimulation have been popular methods to investigate the function of reticulospinal, vestibulospinal, and bulbospinal tracts. In the acoustic startle response, an unexpected acoustic stimulus is delivered, and electromyography (EMG) is recorded from proximal upper extremity muscles to quantify drive from the reticulospinal system (99). The technique has been successfully used in animal models (100–103) and clinical (104–106) studies to identify the involvement of reticulospinal pathways. Using the startle response, it has been suggested that reticulospinal pathways are involved in spasticity (107) and have enhanced function in severely impaired patients (75); although contradictory findings have also been reported (108, 109). A recent review has suggested that stroke heterogeneity likely attributes to outcomes observed with the startle reflex and should be accounted for if deployed in clinical populations (110). It is possible that new methods, though, such as the StretchfMRI may circumvent some concerns noted with the startle reflex alone (28).

Transcranial magnetic stimulation (TMS) has also been used to define surrogate function of the reticulospinal tract (111), but also the vestibulospinal tract. Here, ipsilateral motor evoked potentials (iMEPs) in proximal limbs are used as a measure of reticulospinal tract functional connectivity (80, 112). Using this tool, it has been suggested that following a stroke, reticulospinal tracts have a proximal muscle innervation bias (112, 113). Similar approaches, but instead with galvanic vestibular stimulation, have been used to evaluate the vestibulospinal tract (114–117).

## 4 Moving forward: evaluating motor pathways through novel techniques

Overall, work in pre-clinical and clinical models have demonstrated a significant role of alternate motor pathways in baseline impairment and motor recovery after stroke. We believe that one approach that should be considered to expand knowledge of the structure of alternate motor pathways after stroke is the use of novel neuroimaging approaches in the spinal cord. Specifically, to truly evaluate alternate motor pathways, structures beyond the brain must be considered. In the brain, most ascending and descending pathways overlap and are difficult to distinguish with available neuroimaging techniques. While neurophysiological approaches provide some insight, they remain surrogate markers of the alternate motor pathways. Instead, since many alternate motor pathways originate below the subcortical regions of the brain, areas such as the brain stem and spinal cord would need to be evaluated to provide the most clarity. However, a logical question would be whether changes in pathways can be observed in structures so distant from the original site of the ischemic lesion? Further, would information about pathways in the spinal cord be useful in populations with stroke?

### 4.1 Can we evaluate alternative motor pathways degeneration after stroke in the spinal cord and would it be useful?

The feasibility of identifying changes in alternate motor pathways in such structures post-stroke has been validated by observations in post-mortem studies. Specifically, post-mortem studies have provided initial groundwork showing that pathways within the spinal cord (corticospinal and alternate motor) show atrophy in chronic stroke patients. Of note, several notable studies (3) dating back to the early 1900s, have evaluated post-mortem the cervical and thoracic spinal cords of stroke patients. For example, Buss et al. (118) evaluated post-mortem spinal cords from twenty-six subjects, including four controls and twenty-two post-stroke patients. It was observed that there was a gradual loss of myelin in the spinal cord as early as 1–4 months after the stroke (118). Additionally, astrocytic scarring was detected in cervical sections of the spinal cord at the areas of myelin loss, and changes in the spinal cord after stroke were observed in both the cervical and thoracic regions of the spinal cord, although the timing of these events remains unclear (118). Collectively, post-mortem work has suggested that an insult in the brain may lead to degeneration in the spinal cord and that this degeneration may continue for years after. Thus, post-mortem work has established that changes can be observed in areas caudal of the brain, and therefore it may be possible to study alternate motor pathways in more detail using spinal cord and brainstem imaging techniques (35, 119–129).

Changes in the spinal cord may occur after stroke, but would the knowledge gained be helpful? Certainly. Recent applications have suggested that the spinal cord holds particular potential as a target for neurorehabilitation after stroke. For example, recent work by the Capagrosso group has shown that spinal cord stimulation of the cervical spinal cord in two patients with chronic stroke improved strength, movement and kinematics (130, 131). Specifically, following 19 sessions of spinal cord stimulation of the cervical spinal cord, chronic stroke participants were able to reach areas that were unattainable prior to intervention. This work only supports prior reports in animal models that substantial sprouting could be observed in the spinal cord following constraint induced movement therapy post-stroke (132–134). In fact, stroke animal models have evaluated axonal sprouting in the spinal cord over the past 10–15 years (135, 136), with overall findings suggesting a link to movement and neuroplastic changes in the cortices. We therefore believe that clinical evaluation of similar pathways holds great potential in improving neurorehabilitation approaches post-stroke. For example, identifying and monitoring changes in sprouting and dynamics of alternate motor pathways in the spinal cord may not only improve our clinical pathophysiological understanding of recovery, but also provide insights to help in designing interventions to promote recovery post-stroke.

### 4.2 Ease of evaluation of alternate motor pathways in the spinal cord using diffusion weighted imaging alone

Given that it is feasible to observe degeneration or plasticity changes in the spinal cord after stroke, and that such information could inform neurorehabilitation approaches, what techniques could be used to provide morphological details on alternate motor pathways in real-time post-stroke clinical populations? Or, if a new imaging approach could not be found, could changes in image processing be used to evaluate alternate motor pathways?

**Figure 2:**
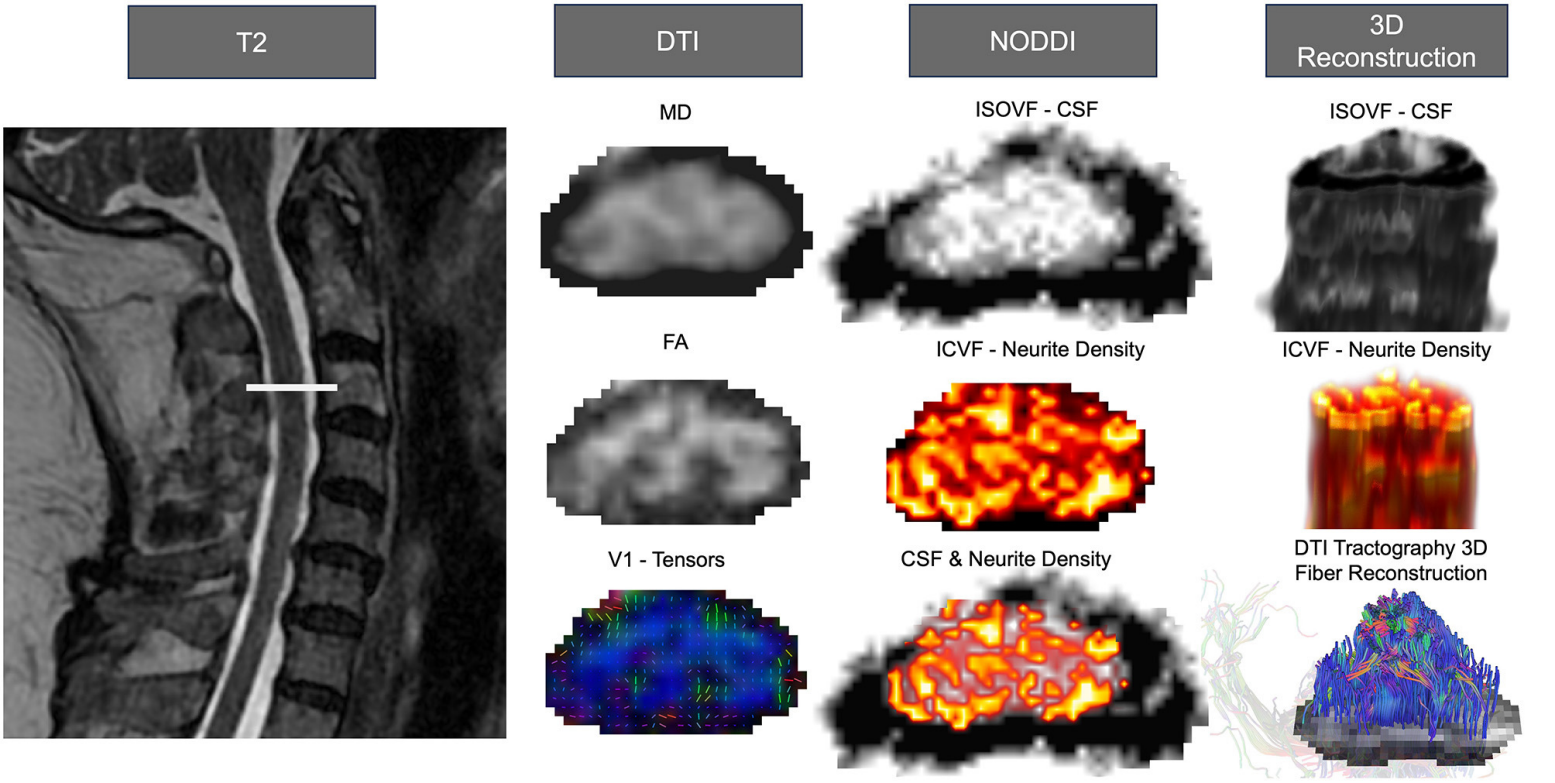
Anatomical tracts, neuroimaging analysis, and 3D reconstruction of the corticospinal tract and alternate motor tracts. Advanced neuroimage processing software, such as FSL for DTI, DSI Studio for DTI Tractography, and AMICO for NODDI, can be employed to quantify diffusion-weighted imaging, determine microstructure properties, and analyze individual tract morphology. All images were from the cervical region and used linear interpolation. The left side depicts the T2 weighted anatomical image with the slice location. The left column shows DTI outputs from SCT processing, MD, FA, and V1. The center illustrates the NODDI processing outputs of isotropic volume fraction (ISOVF; CSF), intra-cellular volume fraction (ICVF; neurite density), and the overlapped representation of neurite density and CSF. On the right are the 3D reconstruction of the CSF **(top)** and neurite density **(center)** visualized using FSLeyes. Additionally, the 3D fiber reconstruction of DTI tractography **(bottom)** is presented using DSIstudio.

Currently a standard approach that is commonly used in stroke populations is diffusion-weighted imaging (DWI). DWI is a technique in magnetic resonance imaging (MRI) used to assess brain and spinal cord white matter (137). Water molecules in the body undergo random translational motion and by applying special diffusion gradients, magnetic resonance can be made sensitive to this motion. For example, DWI allows for the visualization of the ventricles due to free fluid, and the appearance of the brain parenchyma due to a higher level of signal intensity. Diffusion Tensor Imaging (DTI) is another mode of visualization that considers the architecture of axons in parallel bundles and the ability of their myelin to allow for the diffusion of water molecules (Figure 2) (138). By applying diffusion gradients in various directions, a diffusion tensor can be calculated that describes diffusion anisotropy, the concept that water molecule displacement is not equal in all directions (138–144). Anisotropy has been shown to decrease following the occurrence of a lesion (145). Additional insights into white matter morphology post-stroke can be witnessed through the use of three-dimensional fiber tractography (146), and have been used to predict clinical outcomes of stroke patients (147).

DTI at the level of the spinal cord has been suggested to pose challenges, however, due to the range of lesions and difficult imaging conditions around the spinal cord (148). Furthermore, proton relaxation times are very similar for different white matter tracts in the spinal cord making it impossible to differentiate them using DTI alone (149). In addition, as outlined in Figure 1A, many tracts show a spread-out anatomical projection and resolution may be unable to differentiate each alternate motor pathway. Fortunately, software tools, such as DSI Studio (http://dsi-studio.labsolver.org/), FMRIB Software Library (FSL), and the Spinal Cord Toolbox, have been developed to circumvent some limitations of conventional imaging (35, 150, 151). For example, Spinal Cord Toolbox provides a probabilistic reconstruction of the white matter tracts, referencing atlas templates using machine-learning to continuously improve accuracy and reliability. However, although there have been remarkable advances with these tools in tractography, it still remains a relative challenge to study the spinal cord due to the obstacles in image acquisition, post-processing, structural integrity and anatomy, and clinical applications (152). As a result, additional analysis techniques and imaging techniques beyond DTI are needed to assess alternate motor pathways in post-stroke patients.

### 4.3 Moving forward with improved analysis techniques: use of spinal cord toolbox in spinal cord tract analysis combined with DWI

One software that may improve the evaluation of structural and white matter images of the spinal cord following stroke is Spinal Cord Toolbox (SCT) (36). SCT is an open-source software that utilizes Python through command-line or graphical user interface interactions, making integration into many operating systems possible (153). Prior to the release of SCT in 2017, no software package was available to process multi-parametric MRI data in the spinal cord. Thus, SCT represents a novel data processing platform to standardize spinal cord analysis procedures. SCT can process various sequences and contrasts of MRI images (e.g., DWI, T2- and T2^*^- weighted) of the spinal structure (17, 35). The program employs a sequential processing approach to analyze spinal cord MRI data, beginning with T2-weighted images, followed by T2^*^-weighted images, and concluding with diffusion-weighted images (DWI). By utilizing spinal cord MNI-Poly-AMU templates or atlases, the program employs a sophisticated non-linear registration method to align the MRI data from different sequences. This approach ensures accurate and reliable extraction of information about the microstructure and diffusion properties of the spinal cord.

In the context of alternate motor pathways, SCT is advantageous since it can quantify DTI metrics along individual vertebral levels and white matter tracts (Figure 1B). An example of the program's effectiveness is demonstrated in a study conducted by De Leener et al. (119), where T2-weighted images were inputted into the software and successfully used to identify spinal cord cross-sectional area, white/gray matter segmentation, and white matter integrity in various spinal cord pathologies (e.g., multiple sclerosis, amyotrophic lateral sclerosis, syringomyelia, ischemia, and cervical spondylotic myelopathy). Work by De Leener et al. (119) suggests that SCT could potentially be used to track longitudinal changes in neurodegeneration or regeneration.

It should be noted that one key limitation of SCT, however, is that the white matter atlas is built from a single vertebral level (154) and applied to levels superior and inferior using non-linear warping (35, 39). Even though the SCT software regularizes by warping the spinal cord to a template and calculates overall shape and changes in the gray matter, the exact location of the tracts might vary at different levels of the spinal cord and between subjects (35, 36). Another limitation is that the capability of the program is dependent on the quality of the MRI images inputted, for that reason, all images should be visually inspected and excluded if artifacts, low signal, or motion is present. While SCT has a few limitations, it is considered a less biased method in comparison to the current practice of manually drawing the tract of interest based on user knowledge of spinal cord anatomy (35).

In a broader context, there have been over 180 publications using SCT in clinical populations, which we believe provides validation and impetus for use of the toolbox in stroke populations. To date, SCT has only been utilized in three studies in clinical populations with stroke (155). Of note, SCT was recently utilized in a stroke population by Karbasforoushan et al. (17), highlighting the utility of SCT in identifying correlations between changes in white matter integrity of alternate motor pathways and motor impairment severity in individuals with stroke. We argue that this is an area that should be expanded in future research in stroke. Studies from the early 1900s have provided impetus that the spinal cord undergoes degeneration post-stroke, and alternate motor pathways have been implicated in both function and recovery post-stroke. Further, the spinal cord has shown to have potential as a target for neuromodulation approaches post-stroke. We propose that future work should consider implementation of SCT in pathologies of stroke to help expand knowledge of integrity of alternate motor pathways, particularly in acute and sub-acute timeframes, where gaps in knowledge exist.

### 4.4 Moving forward with improved imaging techniques: use of neurite orientation dispersion and density imaging

Since DWI may be challenging in the spinal cord, additional imaging modalities have been identified to provide pathway-specific detail. Neurite Orientation Dispersion and Density Imaging (NODDI) is a multi-compartment diffusion model that is sensitive to white matter microstructure and neurite morphology (126, 156, 157). NODDI is similar to DWI, however it traditionally utilizes more HARDI shells (at least 2), with added multi-directional intermediate b-values (129). NODDI categorizes several components of the neuronal space including the apparent volume of fractions of axons (v_in_), isotropic water (v_iso_), and the dispersion of fibers about a central axis (orientation dispersion index, ODI) (126), additionally, volume fraction of anisotropic intracellular water (*v*_*ic*_) is used to measure regional neurite density, and ODI as a measure of neurite spatial organization (158). As a result, NODDI expands traditional DWI approaches by providing information about neurite density and the orientation/dispersion of axons and dendrites; microstructural information that has been suggested to impact function post-stroke (159) (Figure 2).

NODDI does have some limitations that should be considered. First, most NODDI sequences have long acquisition times and may not be feasible for all studies; although, some MRI machine manufacturers are exploring experimental sequences that use machine learning algorithms to shorten acquisition time (160). Additionally, the NODDI MATLAB Toolbox software (nitrc.org/projects/noddi_toolbox), requires a significant amount of time to compute NODDI, and typically demands expensive high-performance computing resources. Recently, though, further development called Accelerated Microstructure Imaging via Convex Optimization (AMICO) has yielded remarkable acceleration in computational time by order of magnitudes while eliminating the high resource expense (161).

Overall, NODDI has primarily been used in neurodegenerative diseases, such as cervical myelopathy, and multiple sclerosis (MS) (158, 162, 163), in the brain and spinal cord. For example, Zhang et al. (162) was able to use NODDI to correlate with baseline neurologic function in patients with cervical spondylotic myelopathy, where conventional MRI and DWI did not provide significant contributions. In addition, By et al. (126) evaluated the cervical spinal cord in six MS patients using NODDI. The study observed a decrease in NODDI-derived intra-axonal volume fraction (v_in_) in MS patients, signifying a decrease in dendrite and axon density at the site of the lesion (126, 164). To date, however, very few studies have used NODDI in populations with stroke (156, 165–167), and none have evaluated the spinal cord post-stroke; in fact there was a recent call for large rigorous studies to be completed in stroke to help further validate NODDI. We believe that NODDI may provide a method to improve knowledge around alternate motor pathway morphology post-stroke. Animal and clinical studies have provided framework that changes in motor and alternate motor pathways may show microstructural changes related to motor impairment and recovery (32, 168, 169) post-stroke. Expansion of NODDI into the spinal cord, post-stroke, will allow for a deeper understanding of these changes, particularly in alternate motor pathways.

## 5 Discussion and future directions

The amount of sparing in corticospinal pathways in the lesioned hemisphere after stroke has been suggested to influence baseline function and recovery potential. However, current rehabilitation approaches targeting spared corticospinal pathways have had limited efficacy in patient populations, particularly those with severe impairments. Therefore, it is vital to understand what other pathways beyond the corticospinal tract may influence functional recovery to better inform clinical treatment of patients with stroke. Alternative motor pathways likely have a significant role in stroke recovery. This is because many of these pathways also work independently or in tandem with the corticospinal tract to mediate motor and sensory function. While these alternate pathways likely cannot serve as conduits for full functional recovery (see Section 1), they may hold potential for improving proximal upper extremity function, providing insight into spasticity, compensatory patterns, or recovery rates. A possible role of alternative motor pathways, particularly reticulospinal, rubrospinal, and vestibulospinal pathways, in stroke baseline impairment and recovery has been already implicated.

Determining which alternate motor pathway has the most significance in stroke recovery is a daunting task! We believe this task will be greatly aided by utilizing spinal cord imaging and high-definition spinal cord imaging techniques that have yet to gain attention among stroke researchers. Gross postmortem studies have indicated the feasibility of evaluating alternative motor pathways in the brainstem and spinal cord. Existing advanced image modalities in DTI and NODDI, may allow for microstructure details to be collected across the level of the spinal cord in alternate motor pathways. In tandem, analysis techniques, such as the SCT, may provide a unique opportunity for clinical studies to evaluate the time progression of alternate motor pathway degeneration following a stroke. Moving forward, we recommend further use of neuroimaging and neuroimage analysis techniques that have yet to be widely used in stroke populations to expand our knowledge of alternative motor pathways. While image analysis in the spinal cord provides unique challenges i.e., overlapping pathways (Figure 1), pairing existing neurophysiological techniques with new imaging parameters may result in a better baseline understanding of these pathways and the subsequent refinement of rehabilitation practices in stroke survivors.

## Author contributions

RO: Writing—original draft, Writing—review & editing. VC: Writing—original draft, Writing—review & editing. SU: Writing—original draft. SM: Writing—original draft. DS: Writing—original draft, Writing—review & editing. MD: Funding acquisition, Writing—original draft. KP-B: Conceptualization, Formal analysis, Funding acquisition, Project administration, Resources, Supervision, Writing—original draft, Writing—review & editing.
